# Shockpulse lithotripsy during laparoscopic ureterocalicostomy for renal stone concomitant with secondary ureteropelvic junction obstruction: A case report

**DOI:** 10.1016/j.ijscr.2025.110823

**Published:** 2025-01-04

**Authors:** William Tendi, Hendy Mirza

**Affiliations:** a Department of Urology, Cipto Mangunkusumo Hospital, Faculty of Medicine Universitas Indonesia, Jakarta, Indonesia; b Department of Urology, Persahabatan General Hospital, Jakarta, Indonesia

**Keywords:** Case report, Laparoscopy, Nephrolithiasis, ShockPulse lithotripter, UPJO

## Abstract

**Introduction:**

In adult patients, most ureteropelvic junction obstruction (UPJO) occurs secondarily. Concurrent UPJO with nephrolithiasis is not rare and simultaneous treatment by performing laparoscopic pyeloplasty and endoscopic stone removal has been suggested. In the case of atypical anatomy or previously failed pyeloplasty, a laparoscopic ureterocalicostomy is preferred. This study presents a case where a severe UPJO coexistence with renal stone was treated with laparoscopic ureterocalicostomy and direct lithotripsy using ShockPulse lithotripter through the laparoscopic port.

**Presentation of case:**

A 24-year-old male with a history of bilateral open pyelolithotomy came with left flank pain. Further examination revealed a left UPJO and multiple nephrolithiasis. During laparoscopic pyeloplasty, significant adhesion and fibrosis were encountered around the ureteropelvic junction, rendered it challenging to perform pyeloplasty. Consequently, left ureterocalicostomy and direct lithotripsy using ShockPulse lithotripter through the laparoscopic working port were performed. After 3 months, the double-J stent was removed with no complication.

**Discussion:**

The typical minimal invasive options for stone removal for UPJO with large or multiple nephrolithiasis are through percutaneous nephrolithotripsy (PCNL) or direct stone removal during laparoscopy. However, by using the ShockPulse lithotripter, large or multiple calculi can be easily fragmented and siphoned through the laparoscopic port. This technique might help to shorten the operative time and reduce the risk of migrating stone to the abdominal cavity after opening the inferior calyx.

**Conclusion:**

Simultaneous lithotripsy using ShockPulse lithotripter during laparoscopic ureterocalicostomy is a safe and feasible option in treating complex UPJO concurrent with renal stone.

## Highlights

• Laparoscopic ureterocalicostomy and lithotripsy can be performed simultaneosly.
• Possibility of using endoscopic lithotriptor directly through laparoscopic port.
• ShockPulse lithotriptor is able to fragment and siphon calculi simultaneously.
• ShockPulse lithotriptor does not need irrigating fluid.

## 1 Introduction

Ureteropelvic junction obstruction (UPJO) is one of the most common anatomical abnormalities in children presenting with hydronephrosis. Most UPJO are detected early, thus the incidence in adults is rare and the etiology is usually acquired instead of congenital such as external compression on the ureteropelvic junction or an internal abnormality of the ureter such as fibrosis or mass [1,2].

Since UPJO leads to urinary stasis in the pelvicalyceal system, concurrent renal calculus is common. When this occurs, open surgery is recommended as standard treatment for both the pyeloplasty and pyelolithotomy [3]. However, minimally invasive procedures by laparoscopic approach for pyeloplasty in conjunction with percutaneous nephrolithotomy has been suggested [3]. In complex UPJO such as parenchymal thinning due to atypical anatomy or failed pyeloplasty, a ureterocalicostomy was preferred [4]. This case presents the use of a ShockPulse lithotripter directly through laparoscopy port during laparoscopic ureterocalicostomy for eliminating the concurrent renal stone in patient with UPJO at the public hospital. This study was written in accordance with SCARE criteria [5].

## 2 Presentation of case

**Fig. 1:**
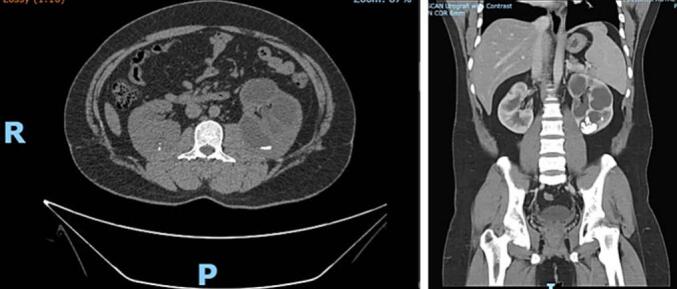
Computed tomography scan of the patient.

**Fig. 2:**
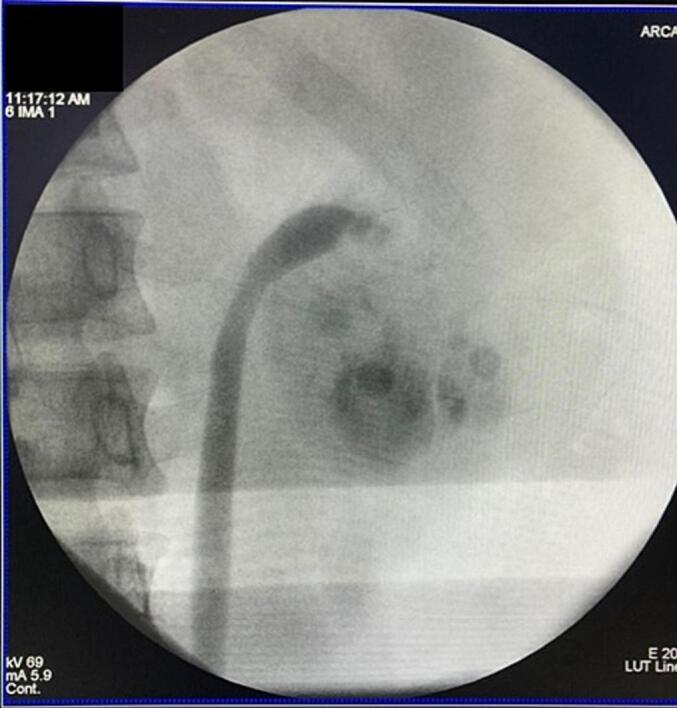
Retrograde pyelogram of the patient.

A 24-year-old male with a history of bilateral open nephrolithotomy presented with left flank pain for 4 months. The pain was intermittent, dull, and not radiating. Further examination revealed normal serum creatinine (1.3 mg/dL) with bilateral hydronephrosis and renal stone (Fig. 1). The stone burden of the right and left kidney was 7 and 29 mm, respectively. The patient was scheduled to have left endoscopic combined intrarenal surgery (ECIRS) at the previous hospital. However, during the left ureteroscopy, severe UPJO was found (Fig. 2), thus the procedure was stopped, and the patient was referred.

**Fig. 3:**
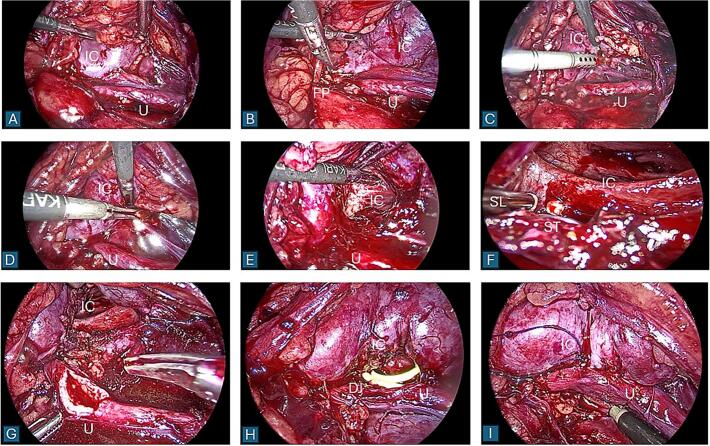
Laparoscopic ureterocalicostomy and lithotripsy under direct vision from laparoscopic scope. DJ = double-J stent; FP = fibrotic pyelum; IC = inferior calyx; SL = ShockPulse lithotripter; ST = stone; U = ureter.

In our hospital, a left nephrostomy tube was placed then the patient was scheduled to undergo transperitoneally laparoscopic left pyeloplasty and stone removal at different setting. However, during the pyeloplasty, after identifying the ureter and inferior calyx (Fig. 3A), there was severe fibrosis and adhesion surrounding the left ureteropelvic junction (UPJ) along with high ureteral insertion (Fig. 3B). Therefore, a ureterocalicostomy was attempted. An incision was made at the inferior calyx, and bleeding was controlled with bipolar cautery (Fig. 3C–E). After the incision, multiple renal stones were encountered. The size of the stones was larger than the laparoscopic trocar. Hence, a lithotripsy was performed using the Olympus ShockPulse-SE Lithotripter, which was guided under direct vision of the laparoscopic scope (Fig. 3F). After all visible stones were removed, ureterocalicostomy was completed (Fig. 3G–I) and the procedure was finished by leaving left nephrostomy tube, left double-J ureteral stent and drain. Total operative time was 4 h with the amount of blood loss was 50 ml.

**Fig. 4:**
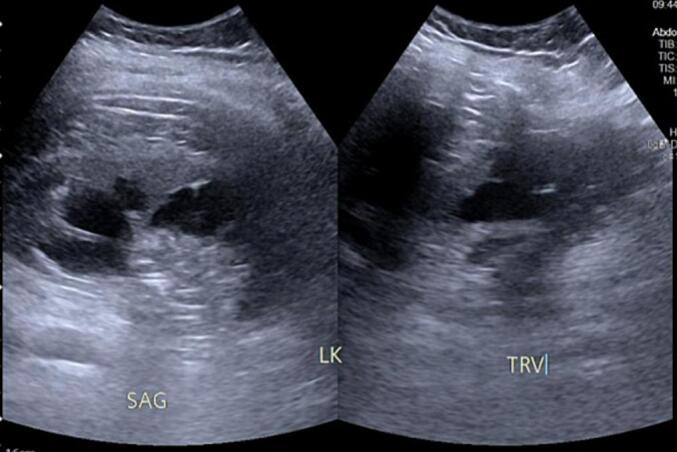
Ultrasound of the patient after 2 months.

**Fig. 5:**
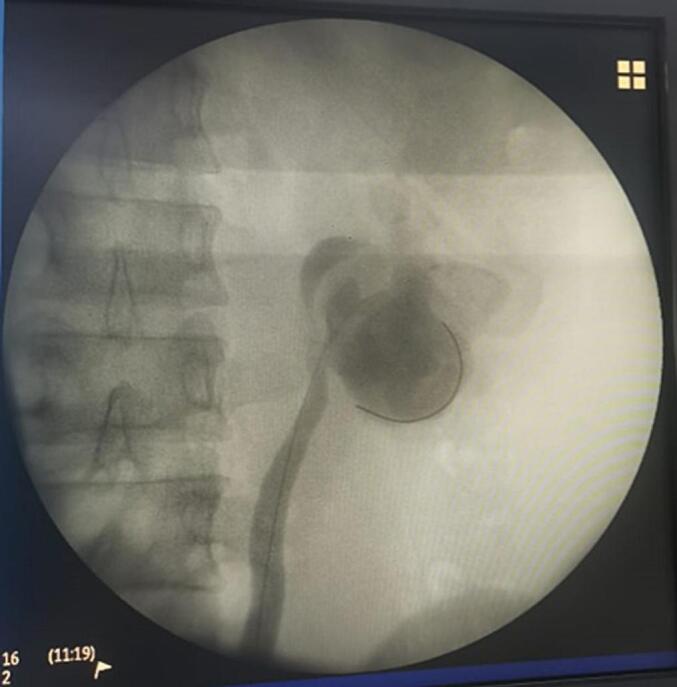
Retrograde pyelogram after 3 months.

The following day after the surgery, the nephrostomy tube was removed. The patient experienced minimal to no pain and was able to walk after 4 days, in which the drain was removed, and the patient was discharged. Evaluation was performed two months later, revealing a significant improvement in left hydronephrosis with no residual stone (Fig. 4). A retrograde pyelogram was performed three months after the procedure showing good anastomosis with no stenosis or leakage (Fig. 5). Thus, the double-J stent was then removed.

## 3 Discussion

The case is that of a 24-year-old male with left UPJO and bilateral nephrolithiasis. In the case of concurrent UPJO and renal stone, simultaneous treatment is recommended [6]. Open pyeloplasty and pyelolithotomy have been described as the gold standard treatment [3]. However, minimal invasive procedures have been documented in many studies. A study by Skolarikos *et al* showed that percutaneous nephrolithotripsy (PCNL) followed by antegrade endopyelotomy can be considered if the renal function is more than 25 % and the grading of hydronephrosis is grade 1 or 2. When the indication of endopyelotomy is carefully assessed, the success rate is up to 64–94 % [7]. In addition, Wei *et al* in 2020 observed that antegrade balloon dilation following PCNL was also effective. The dependent factors were similar with endopyelotomy, where patients with mild or moderate hydronephrosis had a higher success rate than those with higher grade (96 % *vs* 50 %, respectively). Furthermore, patients with moderate or good renal function also had a higher success rate than those with poor renal function (93.1 % *vs* 0 %, respectively) [8].

One of the most popular strategies for managing UPJO is laparoscopic pyeloplasty [9]. The procedure can be performed either with a conventional or robotic approach with a high success rate. Most of the laparoscopic procedures were performed transperitoneally and the mean overall obstruction-free rate and stone-free rate in the laparoscopic approach were 96.18 % and 91.33 % respectively. In this approach, the stones were removed manually with graspers under direct vision of the laparoscopic scope through the laparoscopic port. If the stone size was too large, the stone was inserted into an endoscopic bag and removed through the umbilical port at the end of the procedure [7].

In difficult cases such as giant intra-renal pelvis, anatomical anomalies, or recurrent UPJO with severe scar including those with obliterated UPJ after prior surgery, ureterocalicostomy might be a better choice [4,10]. The procedure is performed transperitoneally, and the ureter is mobilized to the lower pole of the kidney and spatulated. The most dependable inferior calyx is incised and anastomosed to the spatulated ureter, leaving a double J stent and drain tube [4].

A recent comparative study by Esposito *et al* described that laparoscopic ureterocalicostomy yielded a similar success rate with laparoscopic pyeloplasty in children. The study also reported there was a higher complication rate in the laparoscopic ureterocalicostomy group. However, the complication was in Clavien 2 classification and did not require any re-intervention [11].

In adults, the research source in conventional laparoscopic ureterocalicostomy for UPJO is scarce. Ramanitharan *et al* in 2019 observed that robotic laparoscopic ureterocalicostomy in adults for secondary UPJO had satisfying outcomes both clinically and radiologically. Despite the different approach, the study showed that laparoscopic ureterocalicostomy is feasible and safe option for UPJO in adults [10].

In the case where a ureterocalicostomy is preferred, the stone removal becomes challenging. Up until now, there is no literature mentioning laparoscopy ureterocalicostomy performed in conjunction with lithotripsy under direct laparoscopic vision. A case report from Nishimura *et al* described a 10-year-old girl who underwent laparoscopic ureterocalicostomy with the removal of renal stone. However, the stone was directly removed through UPJ incision [12]. Another study conducted by Mi *et al* showed that it was possible to perform laser lithotripsy before pyeloplasty by inserting ureteral access sheath and flexible ureteroscope to renal pelvis after incising the pelvis laparoscopically [13]. However, the most common drawbacks of combining laparoscopy and endourological procedure are the large quantities of irrigating fluid entering abdominal cavity when inserting the endourology instruments through the working channel, and the possibility of lost stones in the abdomen during stone removal. The complication of stone being left in the abdomen is rare and there is no report regarding the lost renal stone in the abdomen yet so far. However, a well-described case about gallstone being left inside the abdominal cavity led to an increased risk of abdominal wall abscess, fistula and fever [7].

In our case, during laparoscopic evaluation, the area of the renal pelvis was stenotic and difficult to access. Since the renal parenchyma at the lower pole was thin, a left ureterocalicostomy was decided. After laparoscopic incision of the lower pole of the kidney, multiple calculi were found and a ShockPulse lithotripter was introduced through the working port and the stones were broken down and removed.

The use of ShockPulse lithotripter under direct laparoscopic vision was ideal in our case because it did not need any irrigating fluid from the usual endoscopic scopes. Therefore, there was no risk of accumulating fluid in the abdominal cavity. In addition, the suction feature of the lithotripter reduced the risk of the stone to be “thrown-out” during lithotripsy process. The broken pieces of calculi were siphoned directly into the device and thus reducing the risk of intraperitoneal stone loss. The simultaneous action of fragmenting and siphoning might also reduce the operating time.

This study described the first reported case of ShockPulse lithotripter utilization during laparoscopic ureterocalicostomy in patients with UPJO and renal calculi. The outcome of this case is a successful stone clearance and obstruction release. Therefore, the use of ShockPulse lithotripter during laparoscopic ureterocalicostomy is a feasible option in treating concomitant renal stone in complex UPJO. However, this technique can only be considered when the stones were located at the inferior calyx with thin parenchymal wall where the anastomosis is planned to be made. In the case of superior or medial calyx stones, the probe might not be able to reach the stones. In those scenarios, the open approach might be a better option.

The limitation of this study is that the exact function of the left kidney, both preoperative and postoperative, has not been evaluated. Due to financial reasons, neither renogram nor contrast CT urography can be performed yet in this patient. However, the serum creatinine was routinely monitored every three months. The most recent result was 1.3 mg/dL, showing that the serum creatinine was relatively stable.

## 4 Conclusion

In the current case, concomitant lithotripsy using ShockPulse lithotripter during laparoscopic ureterocalicostomy is a safe and feasible option in treating complex UPJO and inferior renal stone coexistence even with the stone burden of more than 20 mm. In the case of superior or medial calyx stones, the open approach might be preferrable.

## CRediT authorship contribution statement

WT: Conceptualization, Data curation, Formal analysis, Funding acquisition, Investigation, Methodology, Project administration, Visualization, Writing – original draft.

HM: Conceptualization, Data curation, Formal analysis, Resources, Supervision, Validation, Writing – review and editing.

## Consent

Written informed consent was obtained from the patient for publication of this case report and accompanying images. A copy of the written consent is available for review by the Editor-in-Chief of this journal on request.

## Ethical approval

This study has been approved by the ethical committee in our institution.

## Guarantor

William Tendi

## Funding

This research did not receive any specific grant from funding agencies in the public, commercial, or not-for-profit sectors.

## Registration of research studies

Not applicable.

## Declaration of competing interest

The authors have no conflict of interest to disclose.

## References

[1] Al Aaraj MS, Badreldin AM. Ureteropelvic Junction Obstruction. [Updated 2023 Jul 10]. In: StatPearls [Internet]. Treasure Island (FL): StatPearls Publishing; 2024 Jan-. Available from: https://www.ncbi.nlm.nih.gov/books/NBK560740/. 32809575

[2] O'Sullivan N.J., Anderson S. (2023 Jun). Pelviureteric junction obstruction in adults: A systematic review of the literature. Curr. Urol..

[3] Mi Y., Kang Z., Wang J., Yan L., Zhang J. (2024 Mar 26). Treatment of ureteropelvic junction obstruction in patients with renal calculi via laparoscopic pyeloplasty and flexible vacuum-assisted ureteral access sheath ureteroscopy: a multicenter retrospective observational study. BMC Urol..

[4] Lobo S., Mushtaq I. (2018). Laparoscopic ureterocalicostomy in children: The technique and feasibility. J. Pediatr. Urol..

[5] Sohrabi C., Mathew G., Maria N., Kerwan A., Franchi T., Agha R.A. (2023). The SCARE 2023 guideline: updating consensus Surgical CAse REport (SCARE) guidelines. Int. J. Surg. Lond. Engl..

[6] Stasinou T., Bourdoumis A., Masood J. (2017 Jan-Feb). Forming a stone in pelviureteric junction obstruction: Cause or effect?. Int. Braz. J. Urol..

[7] Skolarikos A., Dellis A., Knoll T. (2015 Feb). Ureteropelvic obstruction and renal stones: etiology and treatment. Urolithiasis.

[8] Wei C, Wang T, Chen S, Ren X, Chen X. Concomitant management of renal calculi and recurrent ureteropelvic junction obstruction with percutaneous nephrolithotomy and antegrade balloon dilation. J. Int. Med. Res. 2020 May;48(5):300060520911252. doi:10.1177/0300060520911252. PMID: 32356681; PMCID: PMC7218938. PMC7218938 32356681

[9] Wahyudi I., Tendi W., Rahman F., Situmorang G.R., Rodjani A. (2021 Aug 10). Minimal Invasive Treatment in Pelvic-Ureteric Junction Obstruction: A Comprehensive Review. Res. Rep. Urol..

[10] Ramanitharan M., Lalgudi Narayanan D., Sreenivasan S.R., Sidhartha K., Mehra K., Rajiv K., Khelge V. (2020 Jan). Outcomes of robot-assisted Ureterocalicostomy in secondary Ureteropelvic junction in adults: initial experience using Da Vinci xi system with near-infrared fluorescence imaging. J. Laparoendosc. Adv. Surg. Tech. A.

[11] Esposito C, Blanc T, Patkowski D, Lopez PJ, Masieri L, Spinoit AF, Escolino M. Laparoscopic and robot-assisted ureterocalicostomy for treatment of primary and recurrent pelvi-ureteric junction obstruction in children: a multicenter comparative study with laparoscopic and robot-assisted Anderson-Hynes pyeloplasty. Int Urol Nephrol. 2022 Oct;54(10):2503–2509. doi:10.1007/s11255-022-03305-2. Epub 2022 Jul 21. PMID: 35861906; PMCID: PMC9463286. PMC9463286 35861906

[12] Nishimura Y., Moriya K., Nakamura M., Kitta T., Kanno Y., Chiba H., Kon M., Shinohara N. (2017 Jul 6). Laparoscopic ureterocalicostomy for ureteropelvic junction obstruction in a 10-year-old female patient: a case report. BMC Res. Notes.

[13] Mi Y., Kang Z., Wang J., Yan L., Zhang J. (2024 Mar 26). Treatment of ureteropelvic junction obstruction in patients with renal calculi via laparoscopic pyeloplasty and flexible vacuum-assisted ureteral access sheath ureteroscopy: a multicenter retrospective observational study. BMC Urol..

